# Kinome-Wide Functional Genomics Screen Reveals a Novel Mechanism of TNFα-Induced Nuclear Accumulation of the HIF-1α Transcription Factor in Cancer Cells

**DOI:** 10.1371/journal.pone.0031270

**Published:** 2012-02-15

**Authors:** Angela Schoolmeesters, Daniel D. Brown, Yuriy Fedorov

**Affiliations:** 1 Thermo Fisher Scientific, Lafayette, Colorado, United States of America; 2 Thermo Fisher Scientific, Pittsburgh, Pennsylvania, United States of America; University of Georgia, United States of America

## Abstract

Hypoxia-inducible factor-1 (HIF-1) and its most important subunit, HIF-1α, plays a central role in tumor progression by regulating genes involved in cancer cell survival, proliferation and metastasis. HIF-1α activity is associated with nuclear accumulation of the transcription factor and regulated by several mechanisms including modulation of protein stability and degradation. Among recent advances are the discoveries that inflammation-induced cytokines and growth factors affect protein accumulation of HIF-1α under normoxia conditions. TNFα, a major pro-inflammatory cytokine that promotes tumorigenesis is known as a stimulator of HIF-1α activity. To improve our understanding of TNFα-mediated regulation of HIF-1α nuclear accumulation we screened a kinase-specific siRNA library using a cell imaging–based HIF-1α-eGFP chimera reporter assay. Interestingly, this systematic analysis determined that depletion of kinases involved in conventional TNFα signaling (IKK/NFκB and JNK pathways) has no detrimental effect on HIF-1α accumulation. On the other hand, depletion of PRKAR2B, ADCK2, TRPM7, and TRIB2 significantly decreases the effect of TNFα on HIF-1α stability in osteosarcoma and prostate cancer cell lines. These newly discovered regulators conveyed their activity through a non-conventional RELB-depended NFκB signaling pathway and regulation of superoxide activity. Taken together our data allow us to conclude that TNFα uses a distinct and complex signaling mechanism to induce accumulation of HIF-1α in cancer cells. In summary, our results illuminate a novel mechanism through which cancer initiation and progression may be promoted by inflammatory cytokines, highlighting new potential avenues for fighting this disease.

## Introduction

Inflammation is a primary defense process against various extracellular stimuli, such as viruses, pathogens, foods, and environmental pollutants. Several studies have shown that tumorigenesis in many cancers is closely associated with chronic inflammation. Abnormal cellular alterations that accompany chronic inflammation such as oxidative stress, gene mutation, epigenetic change, and inflammatory cytokine release are shared with carcinogenic processes, which form a critical cross-link between chronic inflammation and carcinogenesis. Almost 25% of cancers are reported to occur through chronic inflammation-related processes [1], [2]. The pro-inflammatory regulators such as TNFα and other cytokines and their receptor networks seem to play crucial functions in tumorigenesis [3].

Hypoxia-inducible factor-1 (HIF-1) and its most important subunit, HIF-1α, plays a central role in tumor progression by regulating genes involved in cancer cell survival, proliferation and metastasis [4]. HIF-1 is a major component of the oxygen sensing system that governs cellular responses to decreased oxygen availability. The hypoxia inducible transcription factor HIF-1 is a heterodimer composed of the helix-loop-helix-Per-Arnt-Sim (bHLH-PAS) proteins HIF-1α and the aryl hydrocarbon nuclear translocator (ARNT) also known as HIF-1β. Transactivation of HIF-1 transmits a hypoxic signal into a multitude of pathophysiological responses by regulation of numerous target genes [4], [5].

In addition to hypoxia, more recent evidence suggest that HIF-1 can be accumulated and activated during normoxia by growth factors, cytokines and other factors associated with inflammation [5]. Several reports have indicated an important role of TNFα in regulation of HIF-1α stability and activity [6]–[8]. However, details of HIF-1 regulation by TNFα remain unclear.

Here, we describe signaling mechanisms that incite HIF-1α accumulation in response to TNFα. To improve our understanding of HIF-1 regulation by the cytokine, we screened a kinase-specific small interference RNA (siRNA) library using a HIF-1α-eGFP chimera reporter assay under TNFα treatment. This screen determined that depletion of *ADCK2*, *PRKAR2B*, *TRIB2* and *TRPM7* most significantly downregulates nuclear accumulation of HIF-1α in response to the treatment of osteosarcoma cells. Furthermore, our results suggest that this pathway is also present in prostate cancer cells. Surprisingly, the mechanism of regulation of TNFα-elicited HIF-1α accumulation was associated with a non-conventional NFκB signaling pathway and alleviation of superoxide activity. Taken together our data allow us to conclude that TNFα uses a distinct and complex signaling mechanism to induce accumulation of HIF-1α.

## Results

**Figure 1:**
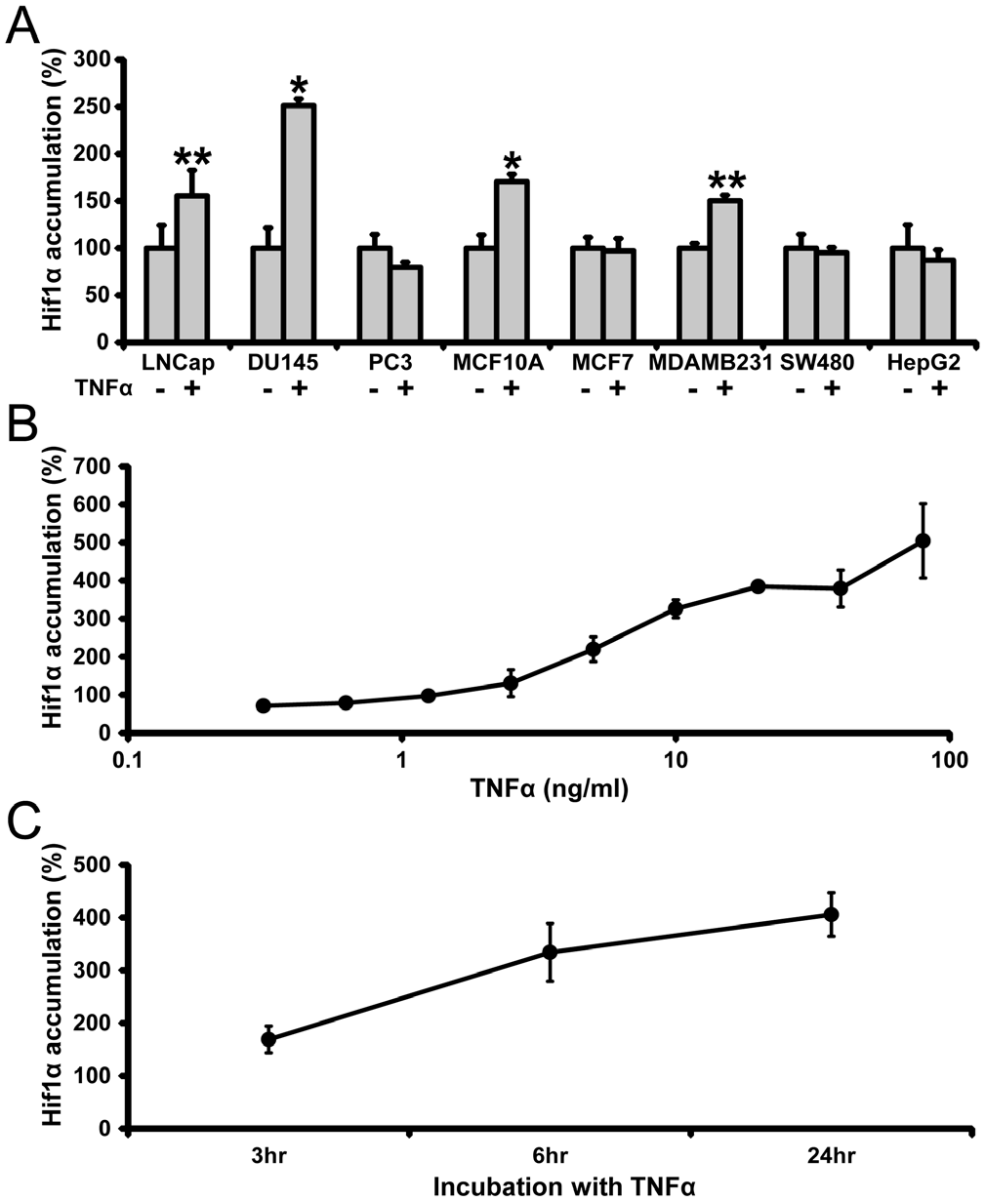
TNFα-induced nuclear accumulation of the HIF-1α transcription factor. (A) TNFα induced nuclear accumulation of HIF-1α in LNCaP, DU145, MCF10a and MDA MB231 cancer cell lines. Expression and nuclear accumulation of HIF-1α was determined by immunocytochemistry imaging as described in Materials and Methods. (B) TNFα-induced stimulation of HIF-1α-eGFP nuclear accumulation in U2OS osteosarcoma in a concentration dependent manner. (C) TNFα stimulated HIF-1α-eGFP nuclear accumulation in U2OS cells in a time-dependent fashion. All data (Median+/−MAD) normalized to untreated cells. For each panel, data are representative of two independent experiments performed in triplicate *, **: Student's t-test p-value between treated cells and corresponding control group, * - p<0.01, ** - p<0.05.

TNFα is a major inflammatory cytokine reported to be a potent inducer of HIF-1α nuclear accumulation [5]–[8]. We examined several cancer cell lines for HIF-1α accumulation under TNFα treatment. In our experiments, TNFα produced a significant increase in nuclear accumulation of HIF-1α in several cancel cell lines (Fig. 1a). Similarly, TNFα induced nuclear buildup of a HIF-1α-eGFP chimera protein (Fig. S1a, Fig. 1b,c) in the HIF-1α_U2OS Redistribution assay based on an osteosarcoma cell line. The observed effect was concentration- and time-dependent (Fig. 1b,c). 24 hr incubation with TNFα at 10 ng/mL was selected for all screening experiments to provide an appropriate window to study up- and down-regulation of HIF-1α accumulation.

**Figure 2:**
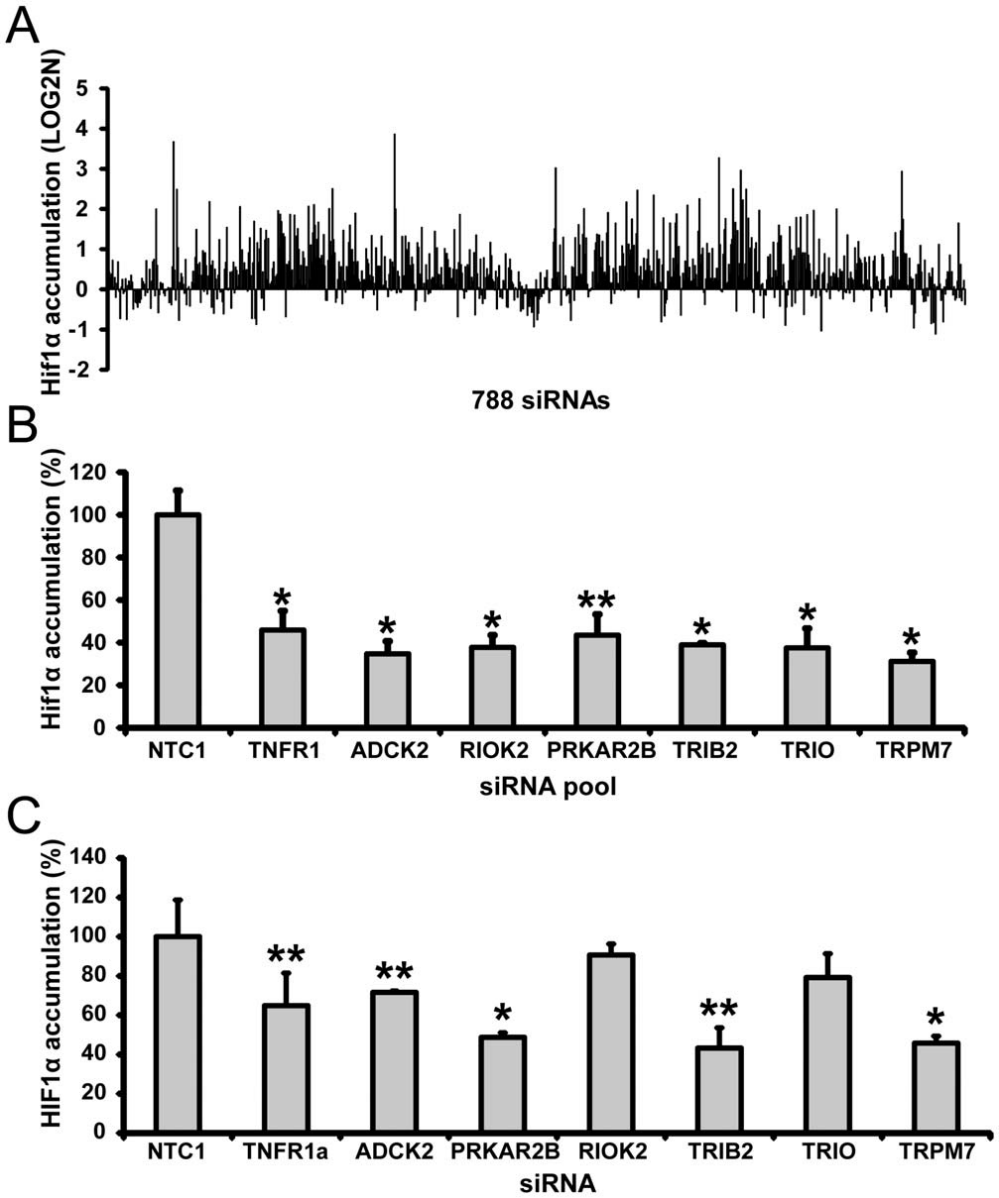
RNAi Screening to identify regulators of TNFα-induced accumulation of HIF-1α. (A) Representative data set from a kinome-wide screen of 788 siRNAs. Data (Median+/−MAD) are representative of three individual transfections and are normalized to control siRNA. (B) Selected siRNA hit candidates were tested in separate experiments. siRNAs targeting *ADCK2, RIOK2, PRKAR2B, TRIB2, TRIO and TRPM7* were confirmed as hit candidates and subjected to further analysis. All data normalized to cells transfected with control siRNA NTC1. (C) Effect of selected siRNA hit candidates on HIF-1α accumulation in LNCaP prostate cancer cell line. Data (Median+/−MAD) are representative of three independent experiments performed in triplicate. All data normalized to TNFα-treated cells. *, **: Student's t-test p-value between treated cells and corresponding control group, * - p<0.01, ** - p<0.05.

There are two receptors described for TNFα, namely TNF receptor 1 (TNFR1, p55 receptor) and TNF receptor 2 (TNFR2, p75 receptor). TNFR1 is ubiquitously expressed while TNFR2 is mainly expressed in immune cells [9]. Although both receptors bind TNFα, the main receptor mediating cellular effects in most cell types is TNFR1. In our experiments, knockdown of the TNFR1 effectively diminished TNFα-dependent nuclear accumulation of HIF-1α (Fig S1b). TNFα is known to activate multiple pathways downstream of TNFR1 [9]. To explore the role of kinases in regulating HIF-1α accumulation under TNFα treatment, we depleted kinases in HIF-1α-U2OS cells using a siRNA kinase library targeting 788 kinases and then analyzed cellular accumulation of HIF-1α-eGFP after incubation with TNFα (Fig. 2a). To minimize siRNA off-target activity we used ON-TARGET*plus* version of the human kinases collection of SMART*pool* siRNA reagents [10]. The same kinase library was screened in a control cell line that expresses only eGFP to subtract possible non-specific effects. HIF-1α-eGFP screening data were subjected to Student t-test p-value analysis, Benjamini-Hochberg multiple comparisons correction [11], and performance ranking followed by comparison between two independent screening experiments with a 1.5 fold change threshold. Resulting data was further compared with data from the counter-screen with cells expressing eGFP only (1.2 fold change threshold for eGFP only, Fig S2) and overlapping hits dismissed. Among the 788 kinases screened by siRNA-mediated silencing, depletion of 77 genes increased HIF-1α-eGFP accumulation above 2 fold (Table S1) and depletion of another seven target genes decreased accumulation under TNFα treatment (Fig. 2a). The genes demonstrating a siRNA-mediated decrease of HIF-1α accumulation were of particular interest because these could potentially represent members of TNFα signaling pathways. These include *PRKAR2B, ADCK2, TRPM7, RIOK2, TRIO, ADRA1B and TRIB2*. To confirm that the decrease in TNFα-induced HIF-1α accumulation in siRNA-transfected cells was directly related to depletion of selected targets we repeated this experiment using newly synthesized siRNA pools (Fig. 2b). Only one out of seven selected hit candidates was not confirmed: siRNA targeting *ADRA1B* (data not shown), which was subsequently omitted from further analysis.

A High Content Analysis approach allows simultaneous acquisition of multiple data streams from the same set of samples. We utilized this approach to further analyze the screening data. Collected data (cell number per field) suggested that none of the selected siRNAs (*PRKAR2B, ADCK2, TRPM7, RIOK2, TRIO, and TRIB2)* produced any effect on cell viability (Fig S3). In addition to the control of protein stability, HIF-1α function can be regulated by processes that influence its subcellular localization, e.g. cytoplasmic vs. nuclear. Simultaneous measurement of HIF-1α accumulation in the nuclei and cytoplasm revealed that none of the siRNA targets selected for a decrease in nuclear accumulation produced an increase in cytoplasmic retention of HIF-1α (data not shown).

**Figure 3:**
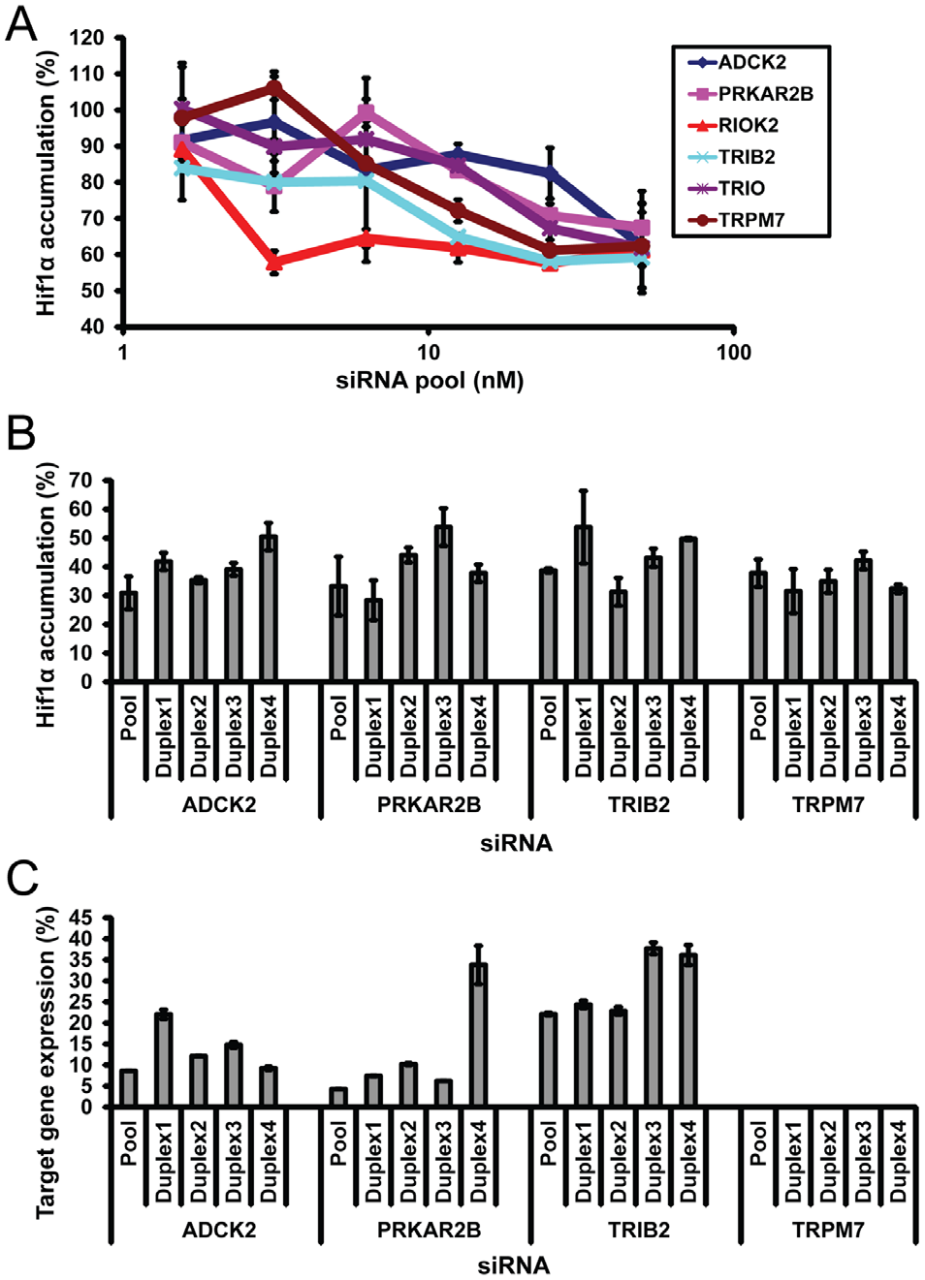
Validation of siRNAs targeting selected hit candidates. (A) Selected siRNAs decrease TNFα-mediated HIF-1α accumulation in a concentration dependent maner. (B) siRNA pools and individual siRNA molecules produced comparable effects on HIF-1α accumulation. (C) siRNA pools and individual siRNA molecules for the selected hit candidates provide effective target gene depletion. U2OS cells were incubated with TNFα for 24 hr. All data normalized to cells transfected with control siRNA NTC1. Data are representative of two independent experiments, three (A,B,Median+/−MAD) or two (C, Mean+/−STDEV) individual transfections each.

To examine if selected candidate hits can influence TNFα-mediated accumulation of HIF-1α in other cell types we determined HIF-1α nuclear buildup in prostate cancer cell lines. HIF-1α accumulation in LNCaP and DU145 cells was found to be sensitive to TNFα while PC3, a prostate cell line with significant invasive potential [12] , demonstrated no such sensitivity (Fig. 1a,). Depletion of *PRKAR2B*, *ADCK2*, *TRPM7* and *TRIB2* significantly decreased HIF-1α accumulation in LNCaP, an androgen-sensitive human prostate adenocarcinoma cell line with low invasive potential (Fig. 2c). Data similar to U2OS and LNCaP were also obtained from MCF10a, a non-tumorigenic mammary epithelial cell line (Fig S4a). In DU145 cells, a prostate cancer cell line with moderate invasive potential, only *TRIB2* depletion was effective in abrogating TNFα-induced HIF-1α accumulation (Fig S4b). Based on the above results *PRKAR2B, ADCK2, TRPM7 and TRIB2* were selected for further analysis. siRNA pools targeting these genes produced concentration-dependent effects on TNFα-stimulated HIF-1α accumulation in U2OS osteosarcoma cells (Fig. 3a)

In all of our experiments we employed strategies that are known to diminish siRNA off-target effects: application of chemically modified siRNA molecules and usage of a siRNA pooling strategy. To further confirm that the decrease in TNFα-induced HIF-1α accumulation in siRNA-transfected cells was directly related to on-target effects of the siRNA, we repeated this experiment using newly synthesized siRNA pools and four separate siRNA duplexes (that comprise each pool) to deplete all four cellular targets. These experiments produced results similar to the screening data – for all four targets siRNA pools and four individual siRNA duplexes produced significant decrease in HIF-1α accumulation (>2 fold, Fig. 3b). Effectiveness of target gene knockdown for selected siRNA hits was determined using Q-PCR and Solaris probes. Target gene depletion correlates with the HIF-1α phenotypical assay - for all four targets siRNA pools used in the screening campaign and four individual siRNA duplexes demonstrated potent knockdown (>60%, Fig. 3c). TRPM7 is poorly expressed in U2OS cells and application of any RNAi reagent effectively eliminated expression of this target to an undetectable level (Fig. 3c).

TNFα is known to regulate expression of proteins within its own signaling cascades. We found that expression of ADCK2 and TRIB2 mRNA is regulated by TNFα in U2OS cancer cells (Fig S5). No statistically significant changes were detected for PRKAR2B and TRPM7.

**Figure 4:**
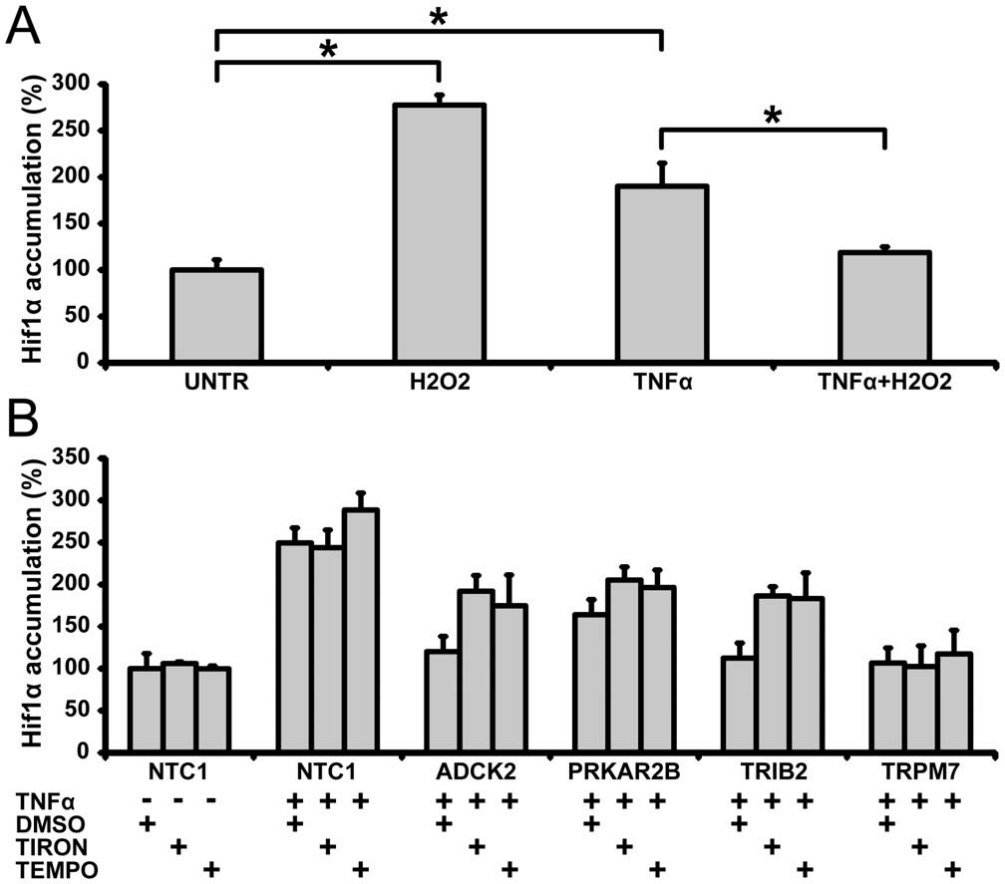
TNFα-induced regulation of HIF-1α is governed by intracellular superoxide accumulation. (A) While addition of hydrogen peroxide (100 µM, 3 hr incubation) can stimulate HIF-1α accumulation, it diminishes such accumulation in TNFα-treated cells (21 hr incubation with TNFα only followed by 3 hr incubation in the presence of both TNFα and hydrogen peroxide). Data (Median+/−MAD) normalized to cells transfected with control siRNA NTC1 and treated with DMSO. (B) Superoxide scavengers rescue HIF-1α accumulation in cells transfected with siRNAs targeting *ADCK2* and *TRIB2* but not *PRKAR2B* or *TRPM7*. Cells were treated with TNFα similar to (A) and incubated with Tiron (0.3 mM), 4-hydroxy-TEMPO (0.1 mM) or DMSO (vehicle control) for 3 hr. Data (Median+/−MAD) normalized to cells transfected with control siRNA NTC1 and treated with DMSO. For each panel data are representative of two independent experiments performed in quadruplicate. *, **: Student's t-test p-value between treated cells and corresponding control group, * - p<0.01, ** - p<0.05.

Recent reports indicate that HIF-1α stability and activity may be regulated through oxidative stress-sensitive pathways [6], [13], [14]. Such pathways are also well known regulators of TNFα signaling [6], [15]. To examine a possible role of oxidative stress mechanisms in TNFα-stimulated HIF-1α accumulation we investigated the effects of exogenous hydrogen peroxide. While hydrogen peroxide alone produced a significant increase in HIF-1α accumulation, an opposite effect was observed on TNFα-pretreated cells (Fig. 4a). This result suggests possible negative regulation of HIF-1α accumulation by superoxide, a main source of intracellular peroxide [16]. We hypothesized that newly discovered positive regulators of HIF-1α accumulation may control either superoxide production or a conversion to peroxide. In U2OS cells, TNFα robustly increased expression of MnSOD, one of the major superoxide/peroxide conversion enzymes (Fig S6). However, depletion of the identified positive regulators of HIF-1α accumulation had no effect on expression of MnSOD (Fig S6).

We observed that superoxide scavengers Tiron and TEMPOL are able to rescue HIF-1α accumulation in TNFα-treated cells transfected with siRNA against ADCK2, and TRIB2 when applied in a concentration that does not significantly affect control cells (Fig. 4b). Cells transfected with PRKAR2B and TRPM7 siRNA were unaffected by superoxide scavengers (Fig. 4b). Taken together our data suggest that TNFα mediates HIF-1α accumulation through a mechanism that mitigates superoxide production, and ADCK2, PRKAR2B and TRIB2 are positive regulators of this mechanism.

Multiple pathways and factors are reported as regulators of HIF-1α activity in other experimental systems and conditions, including conventional NFκB, JNK, STAT3, and proteasome activity [5]. In addition, TNFα is known to induce signaling through the conventional NFκB pathway [9], [17]. Analysis of our results revealed that depletion of kinases that are necessary for these pathways produced no negative impact on TNFα-mediated HIF-1α accumulation. In our experiments, TNFα produced no significant effect on the STAT3-dependent pathway (Fig S7a). We hypothesized that because TNFα induces HIF-1α accumulation, it may also inhibit proteasome activity. To this end we tested TNFα as a possible agonist in the U2OS_E6-AP: p53 degradation and U2OS_SCF-Skp2 E3: p27 degradation Redistribution assays. Proteasome activity inhibitor MG132 induced accumulation of eGFP chimeras in both assays while incubation with TNFα had no effect (Fig S7b,c).

**Figure 5:**
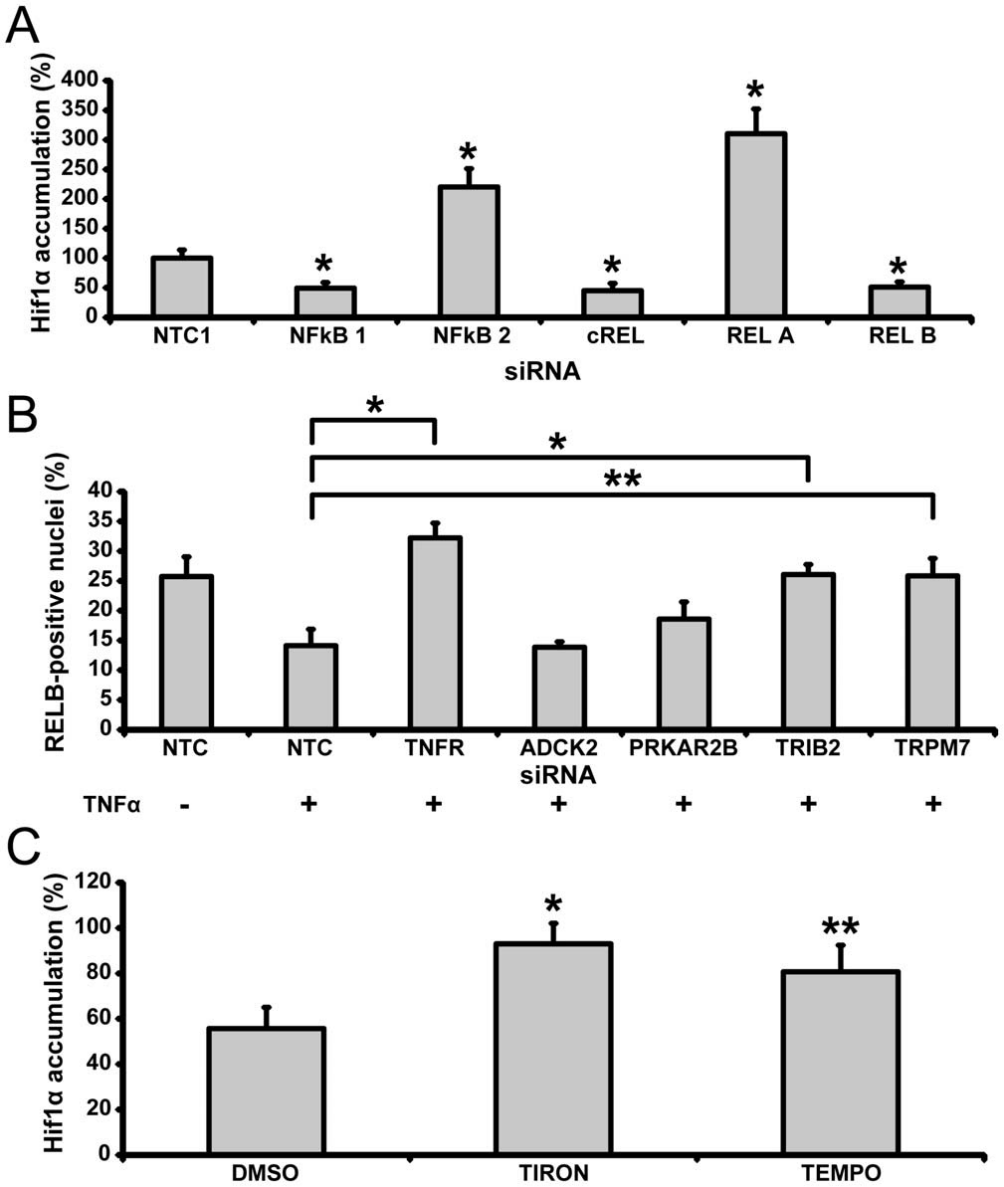
TNFα induces accumulation of HIF-1α through a non-conventional NFκB-dependent pathway. (A) Depletion of NFκB1, RELB and cREL but not NFκB2 and RELA interfere with HIF-1α accumulation in TNFα-treated cells. (B) Depletion of ADCK2 and PRKAR2B prevents accumulation of RELB in the nuclei of TNFα-treated cells. (C) Effect of depletion of RELB is rescued by ROS scavengers. Cells were treated similar to Fig. 4C. (D) All data (Median+/−MAD) is normalized to cells transfected with control siRNA NTC1 treated with TNFα (10 ng/ml, 24 hr). The data are representative of two independent experiments performed in triplicate. *, **: Student's t-test p-value between treated cells and corresponding control group, * - p<0.01, ** - p<0.05.

Data from our screening experiments suggest that depletion of upstream regulators of the JNK pathway results in an increase of HIF-1α accumulation in response to TNFα treatment (Table S1). Depletion of the upstream regulators of the conventional NFκB pathway *CHUK, IKBKB or IKBKE* do not modulate HIF-1α accumulation in response to TNFα treatment (Fig S8). Moreover, siRNA-mediated depletion revealed that NFkB proteins RELA and NFκB2 may act as negative regulators of such accumulation because their knockdown produced sharp increase in HIF-1α accumulation (Fig. 5a). In contrast, NFkB proteins RELB, cREL and NFκB1 appear to be necessary for TNFα-induced HIF-1α accumulation because depletion of corresponding genes produced strong negative effects on accumulation (Fig. 5a). Activation of NFκB proteins correlates with their intracellular translocation [17]. We found that in U2OS osteosarcoma cells, TNFα stimulates translocation of RELB and cREL between the nucleus and cytoplasm, with RELB being excluded from the nucleus and cREL accumulating in the nucleus. Similar to depletion of TNFR1, depletion of TRIB2 and TRPM7 prevented nuclear exclusion of RELB (Fig. 5b). cREL translocation was not affected by depletion of selected targets (data not shown). Furthermore, the effect of RELB depletion was attenuated by superoxide scavengers Tiron and TEMPOL (Fig. 5c). Taken together our results suggest that TNFα-mediated HIF-1α accumulation may be at least partially governed by a non-conventional NFκB signaling pathway activated by TRIB2 and TRPM7.

## Discussion

TNFα is well known to evoke multiple signaling mechanisms where various kinases play irreplaceable roles. Recent advances in functional genomics and cell imaging techniques allowed us to perform systematic investigation of possible mechanisms of TNFα-mediated HIF-1α accumulation. To explore the role of kinases in regulating HIF-1 activity under treatment with TNF-α, we depleted kinases in U2OS osteosarcoma cells using a chemically modified siRNA kinase library targeting 778 kinases and then analyzed nuclear accumulation of HIF-1α-eGFP chimera constitutively expressed in these cells. In this assay, TNFα strongly increased HIF-1α-eGFP protein accumulation (Fig. 1b,c).

Under normal physiological conditions HIF-1α accumulation is heavily repressed by several regulatory pathways. Kinases and related proteins are well known to play an important role in such pathways. Thus, we expected that the majority of siRNA hit candidates would provide a release from the repression of HIF-1α accumulation. Indeed, among the 788 kinases screened by siRNA-mediated silencing, depletion of 6 kinases significantly decreased HIF-1α accumulation and depletion of another 89 kinases increased HIF-1 activity in cells treated with TNFα (Fig. 2a,b and Table S1).

To some extent, our data recapitulates previous findings regarding negative regulators of HIF-1α activity [18]. It was reported that SMG-1 suppresses HIF-1 activity under hypoxic conditions and that siRNA-mediated depletion of the gene product significantly increases activity of a HIF-1α-sensitive reporter [18]. Results of our screening experiments indicate that depletion of SMG-1 specifically up-regulates TNFα-induced HIF-1α accumulation (data not shown).

Several pathways were found to be significantly over-represented in the group of 77 negative regulators: 16 genes represents GO:0007049 cell cycle (*NEK4*, *TAF1L*, *CKS2*, *TTK*, *MAP3K8*, *PIM2*, *TLK1*, *PPP2CA*, *NEK9*, *PRKAG1*, *NEK6*, *PLK4*, *DGKZ*, *DUSP1*, *PIM1*, *PRKAA2*), 11 genes represents GO:0000165 MAPKKK signaling cascade (*PPP2CA*, *RPS6KA3*, *DUSP5*, *MAPKAPK3*, *MAPK8IP3*, *MAP4K5*, *DUSP2*, *MAPK11*, *MAP4K1*, *DUSP1*, *MAP3K9*), and four kinases represent GO:0007254 JNK cascade system (*MAP4K1*, *MAP4K5*, *MAPK8IP3*, *MAP3K9*) (Table S1). These data suggest that such pathways may oppose TNFα signaling and inhibit HIF-1α accumulation.

Depletion of several target genes inhibit TNFα-mediated HIF-1α accumulation (Fig. 2). These genes may represent one or more TNFα signaling mechanisms, and are of particular interest. In our screening campaign we identified six targets of this kind (Fig. 2b). Furthermore, our results suggest that four of these genes - *ADCK2*, *PRKAR2B*, *TRIB2* and *TRPM7* - seem to regulate HIF-1α accumulation in multiple cancer cell lines (Fig. 2, Fig S4). All four genes were previously described in connection with regulation of cancer cell proliferation and motility but existing data did not suggest their participation in TNFα signaling [19]–[23]. The functions of *ADCK2* protein are not yet clear. It is not known if it has protein kinase activity and what type of substrate it would phosphorylate. Several reports established a connection of *ADCK2* to cancer cell proliferation and motility [20]. *PRKAR2B* encodes the cAMP-dependent protein kinase type II-beta regulatory subunit. The cAMP-dependent protein kinase A (PKA) is a ubiquitous serine/threonine protein kinase. PKA is accepted as a major mediator of intracellular cAMP signals in eukaryotes. To date, a large number of cytoplasmic and a few nuclear PKA substrates have been reported [23]. Interestingly, depletion of *PRKAA1* (the catalytic subunit of the PKA) also produced a decrease in HIF-1α accumulation although to a lower extent than *PRKAR2B* depletion (data not shown). Further studies are necessary to clarify the exact role of PKA in TNFα-stimulated HIF-1α accumulation. Although the molecular function of TRIB2 (Tribbles homolog 2) is still unclear, it has been identified as a potential driver of lung tumorigenesis and a myeloid oncogene [21], [22]. TRPM7 is a ubiquitously expressed and constitutively active divalent cation channel. It provides a mechanism for Mg2+ entry and thus it is essential for cell survival and proliferation [19], [24].

**Figure 6:**
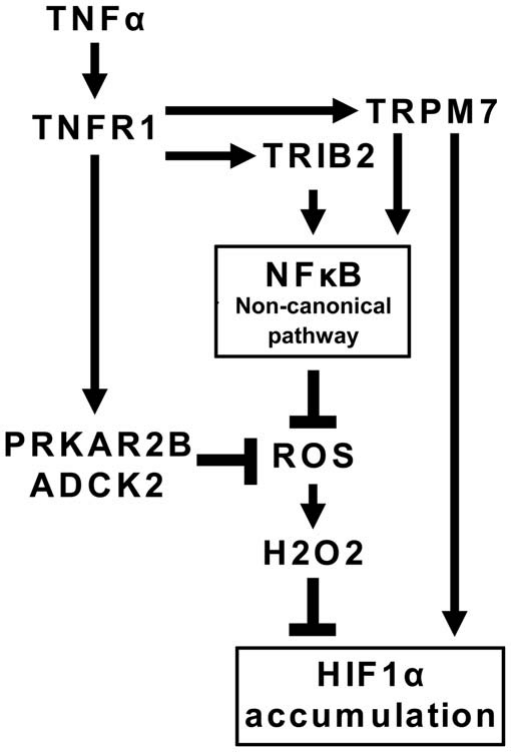
Proposed model for TNFα-induced nuclear accumulation of HIF-1α. Our results suggest that TNFR1 receptor may convey its effect through a complex mechanism that includes a non-conventional NFkB-dependent pathway. This mechanism may negatively regulate production of reactive oxygen species and appears to be controlled by TRIB2, ADCK2 and PRKAR2B.

Key regulators of TNFα signaling pathways are reactive oxygen species (ROS; *e.g*., superoxide, hydrogen peroxide, and hydroxyl radical) [6], [15]. ROS have been suggested to modulate TNFα signaling, providing both positive and negative regulation of the NFκB system downstream of TNFR1 depending on the experimental system and conditions [6], [25]. Our results imply that hydrogen peroxide suppresses TNFα-mediated HIF-1α accumulation (Fig. 4a). These data suggest that the source of intracellular hydrogen peroxide, superoxide anion may inhibit TNFα-mediated HIF-1α accumulation as well. We hypothesized that the newly described regulators of the accumulation may elicit their effect through modulation of superoxide production. Indeed, alleviation of superoxide anion activity rescues HIF-1α accumulation on the background of depletion of ADCK2 and TRIB2 (Fig. 4b). All these results suggest that TNFα-induced HIF-1α accumulation may be regulated by a superoxide sensitive pathway and that the above three proteins may be involved in negative regulation of superoxide production (Fig. 6).

It is well established that conventional and non-conventional NFκB signaling cascades are major mechanisms that convey effects of TNFα on intra-cellular physiology [26]. Our screening results suggest that depletion of positive regulators upstream of conventional NFκB - IKK-α (*CHUK*), IKK-β (*IKKB*) or IKK-ε (*IKBKE*) - produced no negative impact on HIF-1α nuclear accumulation (Fig S8).

Also, we found that depletion of *RELA* and *NFKB2* results in a significant upregulation of HIF-1α accumulation while depletion of *RELB*, *cREL* and *NFKB1* produces a decrease in HIF-1α accumulation. Such a decrease can be rescued by mitigation of superoxide anion activity (Fig. 5). Furthermore, depletion *TRIB2* and *TRPM7* was found to prevent intracellular translocation of RELB upon treatment with TNFα. These findings allowed us to speculate that TNFα may regulate HIF-1α accumulation through both conventional and non-conventional NFκB pathways. The actual amount of accumulated HIF-1α will then depend on a balance between different TNFα-induced NFκB pathways. The proposed model seems to be in line with the known complexity of inflammation-cancer relationships [3].

Taken together our results suggest that TNFα-induced HIF-1α buildup is regulated by a several pathways (Fig. 6). At least in part, TNFα may convey its effect through TNFR1 receptor signaling leading to a non-conventional NFκB-dependent mechanism that negatively regulates production of reactive oxygen species. This mechanism appears to be controlled by ADCK2 and TRIB2. TRPM7 appears to stimulate RELB translocation, but its depletion phenotype can not be rescued by alleviation of superoxide activity. Thus TRPM7 may represent an independent pathway of regulation of HIF-1α accumulation. Further studies are necessary to understand the greater complexity of TNFα-dependent stimulation of HIF-1α nuclear accumulation and its role in tumorigenesis and tumor progression.

## Materials and Methods

### Cell lines

PC3, DU145, LNCaP, MCF10a, MDAMB-231, MCF7, SW480 and HepG2 were obtained from ATCC and maintained according to recommended protocols.

HIF-1α_U2OS Redistribution assay was used in screening campaign to monitor HIF-1α nuclear accumulation: recombinant U2OS cells stably expressing human *HIF-1α* (NM_001530) fused to the C-terminus of enhanced green fluorescent protein (eGFP). U2OS cells are adherent epithelial cells derived from human osteosarcoma. Expression of eGFP-HIF-1α is controlled by a standard CMV promoter and continuous expression is maintained by addition of G418 to the culture medium according to manufacturer protocol. U2OS stable cell line that expresses eGFP only was used as a subtraction control in screening campaign.

In addition to HIF-1α_U2OS the effect of TNFα treatment was tested in three separate Thermo Fisher Scientific Redistribution Assays: the STAT3_U2OS Redistribution Assay, the E6-AP: p53 degradation Redistribution Assay (U2OS), and the SCF-Skp2 E3 Ligase: p27 degradation Redistribution Assay (U2OS). Assays were performed according to manufacturer protocols for all reference compounds. For TNFα-treatment, each assay cell line was treated for 24 hours at 37°C with TNFα at the following concentrations: 80 ng/mL, 40 ng/mL, 20 ng/mL, 10 ng/mL, 5 ng/mL, 2.5 ng/mL, 1.25 ng/mL, and 0.63 ng/mL. Each TNFα titration was performed in quadruplicate on 96-well assay plates, and each assay plate was performed in triplicate. After TNFα treatment, plates were fixed, stained with Hoechst 33258, and imaged as described below. TNFα was purchased from R&D Systems, hydrogen peroxide, Hoechst 33258, Tiron and 4-hydroxy-TEMPO (TEMPOL) were purchased from Thermo Fisher Scientific.

### Transfection

All cell lines were transfected with Dharmafect (DF) tranfection reagents (Thermo Fisher Scientific): DF1 (HepG2, MCF7, MCF10A), DF2 (SW480), DF3 (HIF-1α_U2OS, DU145, LNCaP, PC3) and DF4 (MDA-MB-231). The screening campaign in HIF-1α_U2OS and control cells was performed using an ON-TARGET*plus* version of the human kinases collection of SMART*pool* siRNA regents kinase library targeting 788 kinases (Thermo Fisher Scientific). ON-TARGET*plus* Non-Targeting Control 1(NTC1) siRNA pool was used as a negative control and for normalization purposes. siRNAs targeting TNFR1 and HIF-1α were used as positive controls for transfection efficiency during screening campaign and other transfection experiments.

### Immunocytochemistry

Cultures grown and treated on 96-well plates were fixed in a 4% PFA solution containing 1× Hoechst 33258 (Thermo Fisher Scientific) for 15 min, followed by two washes with PBS. Plates were incubated overnight at 4°C in 1× Blocking Buffer (Thermo Fisher Scientific) diluted in PBS, followed by incubation with primary antibodies diluted in 1× Blocking Buffer for 1 h at RT. Primary antibodies and dilutions were as follows: anti-RelA/p65 (Santa Cruz), 1∶50; anti-RelB (R&D Systems), 1∶40; anti-cRel (Cell Signaling Technologies), 1∶50, anti NFκB1/p105 (Abcam), 1∶200. Plates were washed twice with PBS, followed by incubation with secondary antibody diluted in 1× Blocking Buffer for 1 hour at room temperature. Secondary antibodies and dilutions were as follows: Goat Anti-Mouse IgG (H+L), DyLight 649 Conjugated (Thermo Fisher Scientific, MA), 1∶200; Goat Anti-Rabbit IgG (H+L), DyLight 649 Conjugated (Thermo Fisher Scientific, MA), 1∶200. Plates were washed three times with PBS and sealed for imaging. HIF-1α and MnSOD were detected using the Cellomics HIF-1α and MnSOD Induction HCS Reagent Kits (Thermo Fisher Scientific) according to manufacturer's instructions.

### Cell Imaging

Imaging of harvested cells was performed using the ArrayScan® VTI HCS Reader and CellInsight™ Personal Cell Imager (Thermo Fisher Scientific). eGFP fluorescence was analyzed using the Molecular Translocation BioApplication (Thermo Fisher Scientific). Immunofluorescence was analysed using the Compartmental Analysis BioApplication (Thermo Fisher Scientific). Images were acquired using a 10× objective. Images and data were collected for three fields per well.

### Gene expression analysis

The SV 96 Total RNA Isolation System (Promega, Madison, WI, catalog #Z3505) was used for total RNA purification. The Nanodrop (Thermo Fisher Scientific) was used to determine average concentration of the RNA preps. After isolation, total RNA was frozen at −80°C for storage until further use. Total RNA was thawed once for the RT step, which 5 µL of RNA was used in all cDNA reactions. Verso cDNA Synthesis Kit (Thermo Scientific, catalog #AB-1453) was used for the cDNA synthesis step. cDNA reactions were set up according to the supplier's protocol in a total of 20 µL reactions. Random hexamers and oligo-dT primers in a ratio of 3 to 1 were used in cDNA reactions. Cycling conditions were 42°C for 30 minutes then an inactivation step of 95°C for 2 minutes. No reverse transcriptase enzyme and no template controls were used for each RT run and each gene assay to determine presence of contamination, which all came up negative. Expression of ADCK2 (NM_052853), PRKAR2B (NM_002736), RIOK2 (NM_018343), TRIB2 (NM_021643, TRIO (NM_007118), and TRPM7 (NM_017672) was determined by qRT-PCR in both TNFa treated and untreated cells and transfected cells. Corresponding Thermo Scientific Solaris Human qPCR Gene Expression Assays were purchased from Thermo Scientific - ADCK2 #AX-005304-00, PRKAR2B #AX-007673-00, RIOK2 #AX-005002-00, TRIB2 #AX-005391-00, TRIO #AX-005047-00, and TRPM7 #AX-005393-00. PPIB Solaris Human qPCR Gene Expression Assay (catalog #AX-004606-00) was used to determine PPIB (NM_000942) expression for sample input normalization and analysis of relative expression of the specific genes. All Solaris assays listed do not span an exon-exon boundary or map to any pseudogenes. Either the assay probes or primers cross a splice site. cDNA was diluted 3-fold, and 3 µL of the dilution was the cDNA input for the qPCR reactions. The Thermo Scientific Solaris qPCR Gene Expression Master Mix (Thermo Scientific, catalog #AB-4350) was used for the qPCR step for a 15 µL final reaction volume, and the Ct values used for further analysis were obtained using the Roche Light Cycler 480 (Roche). All qPCR reactions were set up according to the supplier's protocol (1 cycle at 95°C for 15 minutes, then 95°C for 15 seconds followed by 60°C for 1 minute for 40 cycles). In all transfection experiments, relative gene expression was normalized to ON-TARGET*plus* Non-Targeting Pool transfected siRNA controls (Thermo Scientific) for each plate separately. All treatments were tested in biological triplicates. Additional information is presented in Table S2.

### Gene Ontology analysis

L2L (University of Washington) and DAVID (NIAID) tools were used to analyze RNAi screening data.

## Supporting Information

Figure S1 **Effect of TNFα and transfection controls on HIF-1α-EGFP translocation in U2OS osteosarcoma cells.** (A) Exemplary images of HIF-1α _EGFP translocation in control- and TNFα-treated U2OS cells. (B) Effect of TNFα and transfection controls on HIF-1α -EGFP translocation in U2OS osteosarcoma cells. All data (Median+/−MAD) normalized to cells transfected with control siRNA NTC1. Data are representative of three independent experiments, six individual transfections each. *, **: Student's t-test p-value between treated cells and corresponding control group, * - p<0.01, ** - p<0.05.
(TIF)

Figure S2 **Schematic description of selection of hit candidates for positive regulation of HIF-1α accumulation.** Screening data were subjected to Student t-test p-value analysis followed by Benjamini-Hochberg multiple comparisons correction, and performance ranking (top 10% selected). This analysis was followed by comparison between two independent screening experiments. Resulting data were further compared with data from the counter-screen with cells expressing eGFP only. Finally, only hits demonstrating fold change above 1.5 fold were selected for further experiments.
(TIF)

Figure S3 **Effect of selected siRNAs on cell viability of U2OS osteosarcoma cells treated with TNFα.** siRNA targeting *ADCK2, RIOK2, PRKAR2B, TRIB2, TRIO and TRPM7* were transfected into U2OS osteosarcoma cell line. Cells were treated with TNFα (10 ng/mL) for 24 hr before harvesting. Cell number per field was determined 72 hr after transfection. PLK1 siRNA was used as positive control. All data normalized to cells transfected with control siRNA NTC1. Data (Median+/−MAD) are representative of two independent experiments performed in triplicate. All data normalized to TNFα-treated cells.
(TIF)

Figure S4 **Selected siRNAs decrease accumulation of HIF-1α in MCF10a, and DU145 cells incubated with TNFα.** (A) Effect of selected siRNA hit candidates on HIF-1α accumulation in MCF10a breast epithelial cell line. (B) Effect of selected siRNA hit candidates on HIF-1α accumulation in DU145 prostate cancer cell line. Data (Median+/−MAD) are representative of two independent experiments performed in triplicate. All data normalized to TNFα-treated cells.
(TIF)

Figure S5 **Effect of TNFα on expression of selected target genes in U2OS osteosarcoma cells.** U2OS cells were incubated with TNFα for 24 hr. All data (Median+/−MAD) normalized to cells transfected with control siRNA NTC1. Data are representative of two independent experiments, two individual transfections each.
(TIF)

Figure S6 **Effect of selected siRNAs on expression of MnSOD in U2OS osteosarcoma cells treated with TNFα.** siRNA targeting *ADCK2, RIOK2, PRKAR2B, TRIB2, TRIO and TRPM7* were transfected into the U2OS osteosarcoma cell line. Cells were harvested 72 hr after transfection. Cells were treated with TNFα (10 ng/mL) for 24 hr before harvesting. All data normalized to untreated cells transfected with control siRNA NTC1. All data (Median+/−MAD) normalized to cells transfected with control siRNA NTC1.
(TIF)

Figure S7 **Effect of selected siRNAs on nuclear translocation of STAT3, degradation of p27, and degradation of p53 in U2OS osteosarcoma cells.** Cells were incubated with TNFα (10 ng/mL) for 24 hr. Nuclear translocation of STAT3 (A), degradation of p27 (B), and degradation of p53 (C) were determined as described in Materials and Methods. All data (Median+/−MAD) normalized to cells transfected with control siRNA NTC1.
(TIF)

Figure S8 **Effect of CHUK, IKBKB and IKBKE siRNAs on HIF1a accumulation in U2OS osteosarcoma cells.** siRNA targeting CHUK, IKBKB and IKBKE were transfected into U2OS osteosarcoma cells. Cells were harvested 72 hr after transfection. Cells were treated with TNFα (10 ng/mL) for 24 hr before harvesting. All data normalized to untreated cells transfected with control siRNA NTC1. All data normalized to cells transfected with control siRNA NTC1. Data (Median+/−MAD) are representative of two independent experiments performed in triplicate. All data normalized to TNFα-treated cells.
(TIF)

Table S1 **RNAi-based screening identified negative regulators of TNFα-mediated accumulation of HIF-1α.** Fold change values indicate increase in HIF-1α accumulation under TNFα treatment in cells transfected with corresponding siRNA relative to NTC1 control siRNA.
(XLS)

Table S2 **Additional information on Solaris RT-qPCR assay.**
(XLS)
