# Supplementary figures and images for: Kinome-Wide Functional Genomics Screen Reveals a Novel Mechanism of TNFα-Induced Nuclear Accumulation of the HIF-1α Transcription Factor in Cancer Cells
Source: PLoS One. 2012 Feb 15;7(2):e31270. doi: 10.1371/journal.pone.0031270 (PMC3280275; doi:10.1371/journal.pone.0031270)

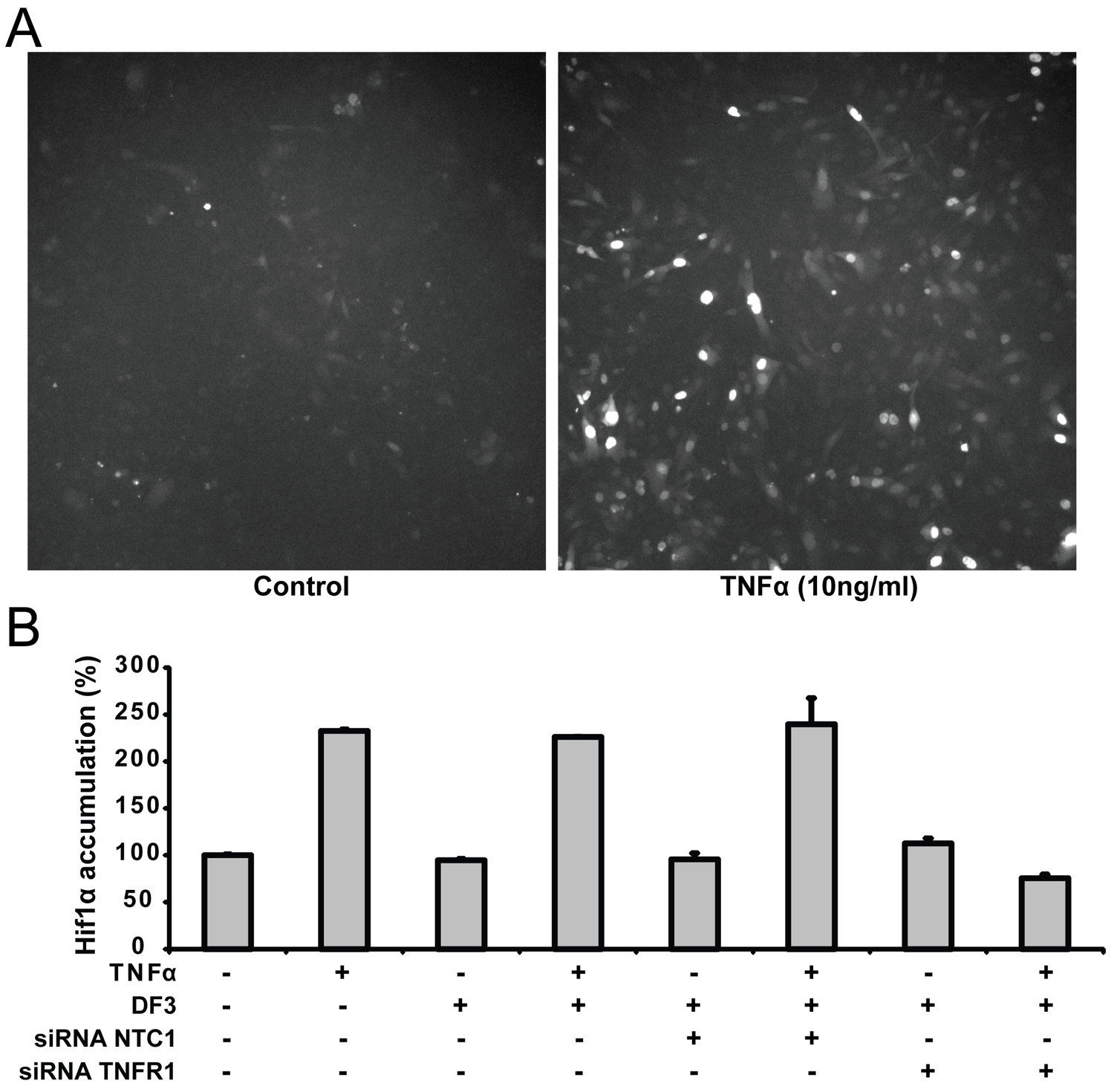

Supplement: Figure S1 — Effect of TNFα and transfection controls on HIF-1α-EGFP translocation in U2OS osteosarcoma cells. (A) Exemplary images of HIF-1α _EGFP translocation in control- and TNFα-treated U2OS cells. (B) Effect of TNFα and transfection controls on HIF-1α -EGFP translocation in U2OS osteosarcoma cells. All data (Median+/−MAD) normalized to cells transfected with control siRNA NTC1. Data are representative of three independent experiments, six individual transfections each. *, **: Student's t-test p-value between treated cells and corresponding control group, * - p<0.01, ** - p<0.05. (TIF) [file pone.0031270.s001.tif]

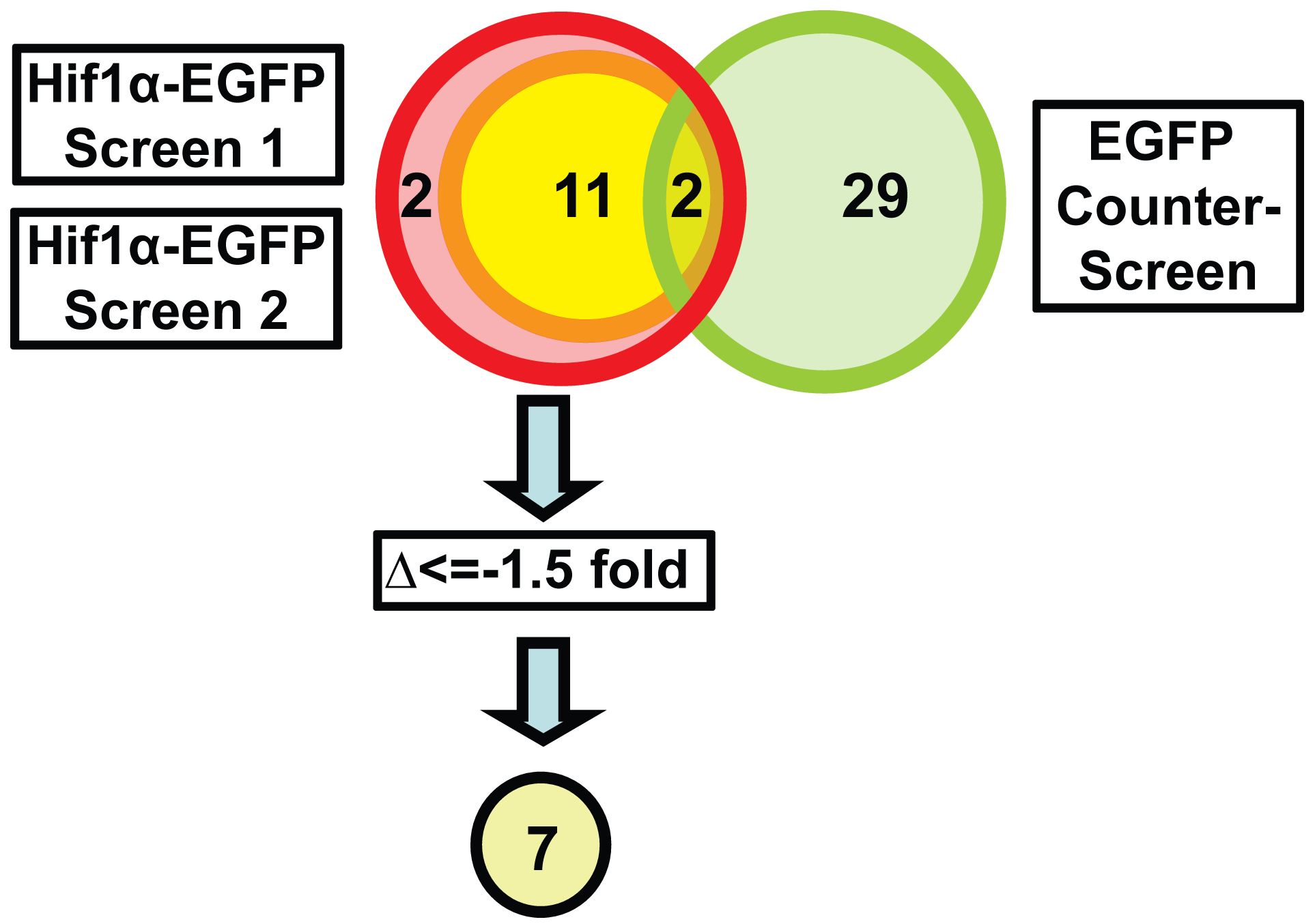

Supplement: Figure S2 — Schematic description of selection of hit candidates for positive regulation of HIF-1α accumulation. Screening data were subjected to Student t-test p-value analysis followed by Benjamini-Hochberg multiple comparisons correction, and performance ranking (top 10% selected). This analysis was followed by comparison between two independent screening experiments. Resulting data were further compared with data from the counter-screen with cells expressing eGFP only. Finally, only hits demonstrating fold change above 1.5 fold were selected for further experiments. (TIF) [file pone.0031270.s002.tif]

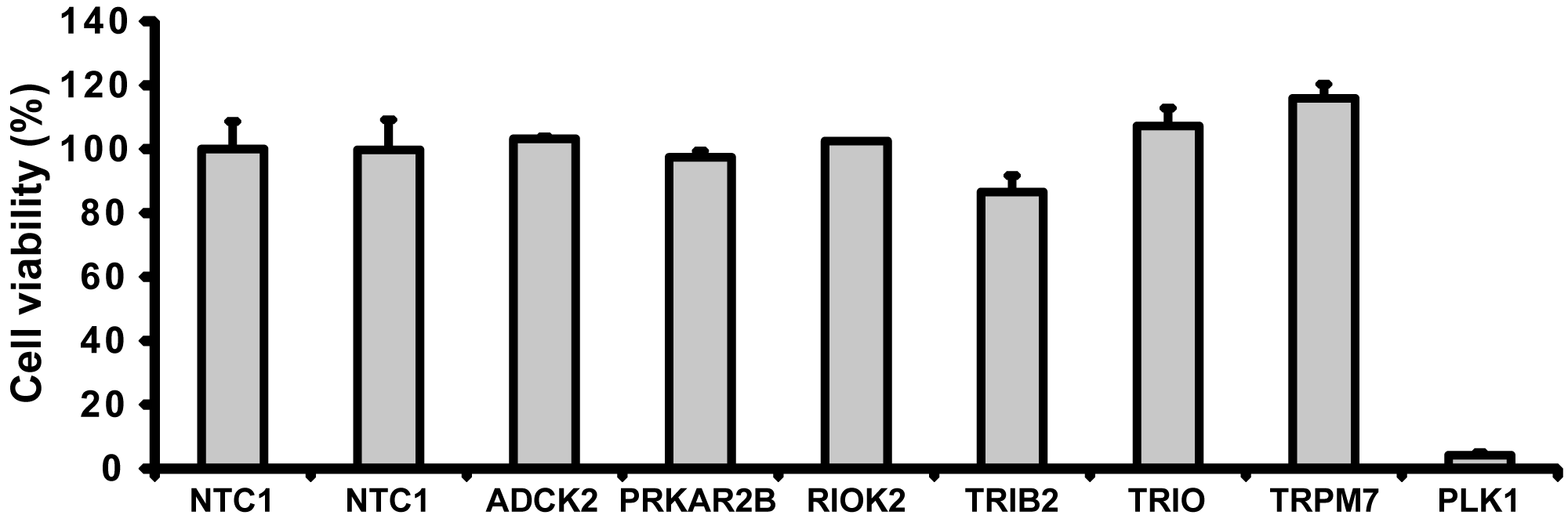

Supplement: Figure S3 — Effect of selected siRNAs on cell viability of U2OS osteosarcoma cells treated with TNFα. siRNA targeting ADCK2, RIOK2, PRKAR2B, TRIB2, TRIO and TRPM7 were transfected into U2OS osteosarcoma cell line. Cells were treated with TNFα (10 ng/mL) for 24 hr before harvesting. Cell number per field was determined 72 hr after transfection. PLK1 siRNA was used as positive control. All data normalized to cells transfected with control siRNA NTC1. Data (Median+/−MAD) are representative of two independent experiments performed in triplicate. All data normalized to TNFα-treated cells. (TIF) [file pone.0031270.s003.tif]

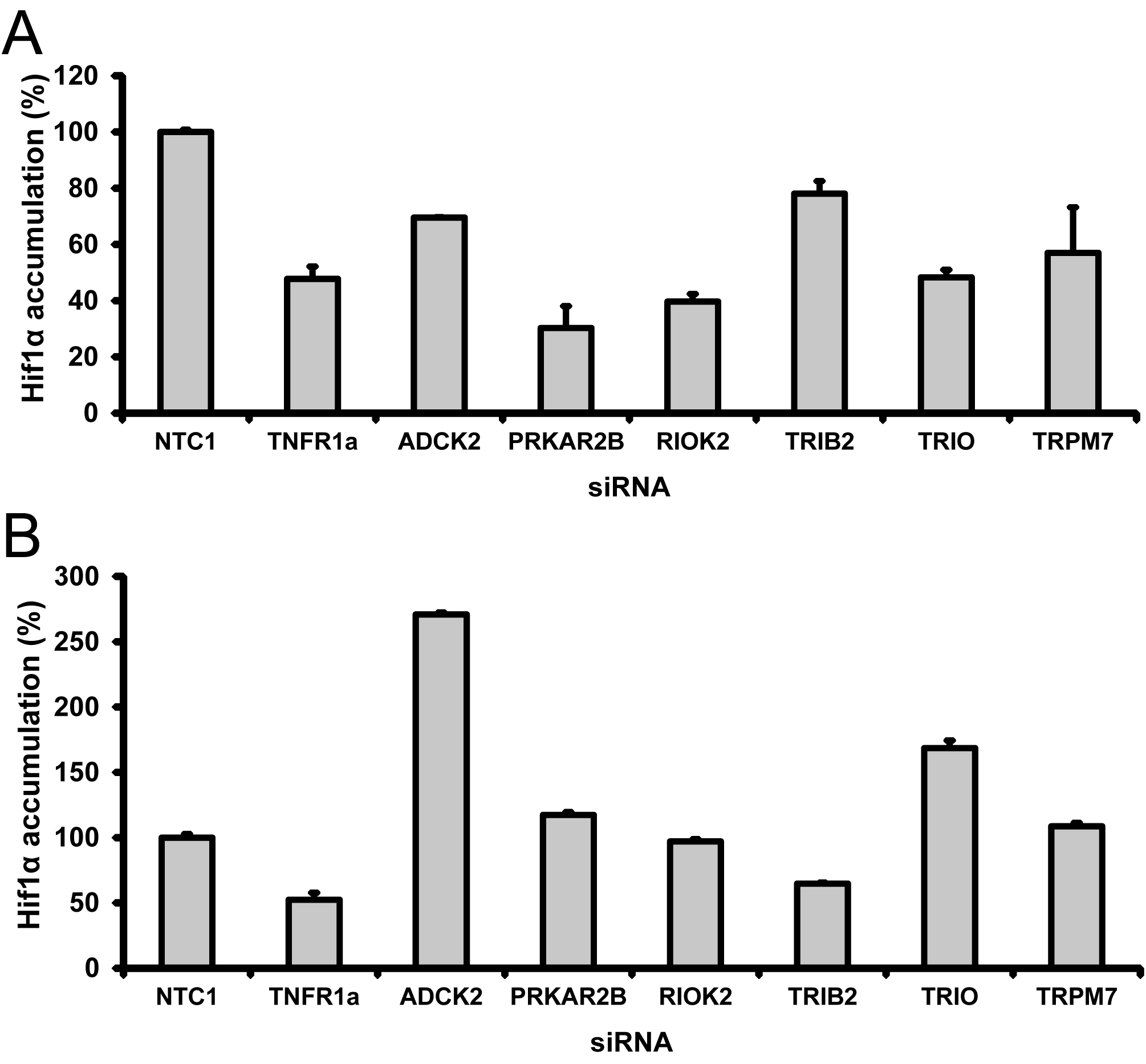

Supplement: Figure S4 — Selected siRNAs decrease accumulation of HIF-1α in MCF10a, and DU145 cells incubated with TNFα. (A) Effect of selected siRNA hit candidates on HIF-1α accumulation in MCF10a breast epithelial cell line. (B) Effect of selected siRNA hit candidates on HIF-1α accumulation in DU145 prostate cancer cell line. Data (Median+/−MAD) are representative of two independent experiments performed in triplicate. All data normalized to TNFα-treated cells. (TIF) [file pone.0031270.s004.tif]

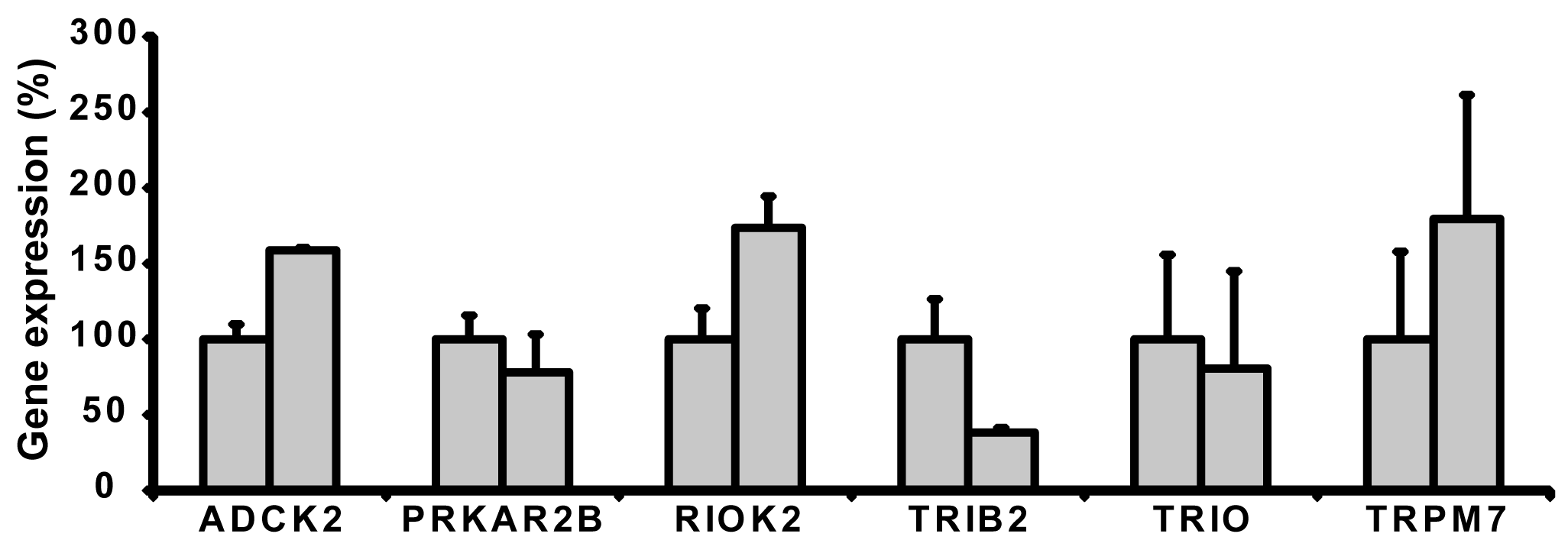

Supplement: Figure S5 — Effect of TNFα on expression of selected target genes in U2OS osteosarcoma cells. U2OS cells were incubated with TNFα for 24 hr. All data (Median+/−MAD) normalized to cells transfected with control siRNA NTC1. Data are representative of two independent experiments, two individual transfections each. (TIF) [file pone.0031270.s005.tif]

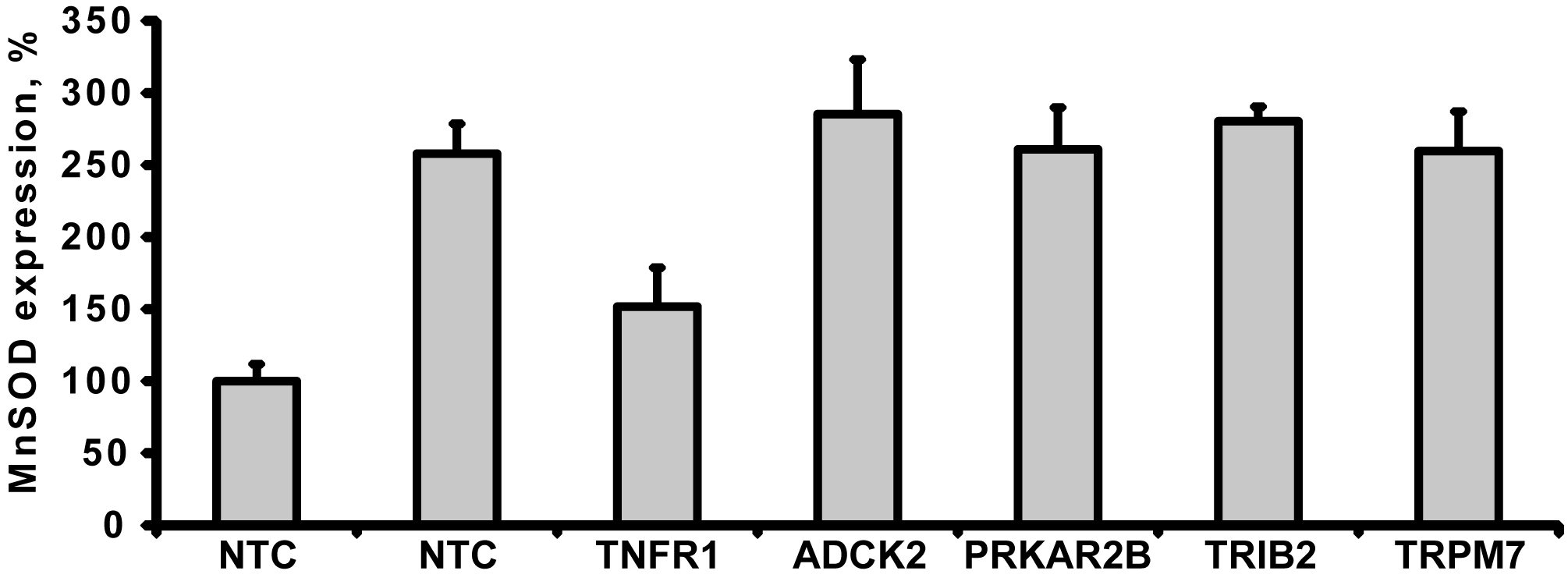

Supplement: Figure S6 — Effect of selected siRNAs on expression of MnSOD in U2OS osteosarcoma cells treated with TNFα. siRNA targeting ADCK2, RIOK2, PRKAR2B, TRIB2, TRIO and TRPM7 were transfected into the U2OS osteosarcoma cell line. Cells were harvested 72 hr after transfection. Cells were treated with TNFα (10 ng/mL) for 24 hr before harvesting. All data normalized to untreated cells transfected with control siRNA NTC1. All data (Median+/−MAD) normalized to cells transfected with control siRNA NTC1. (TIF) [file pone.0031270.s006.tif]

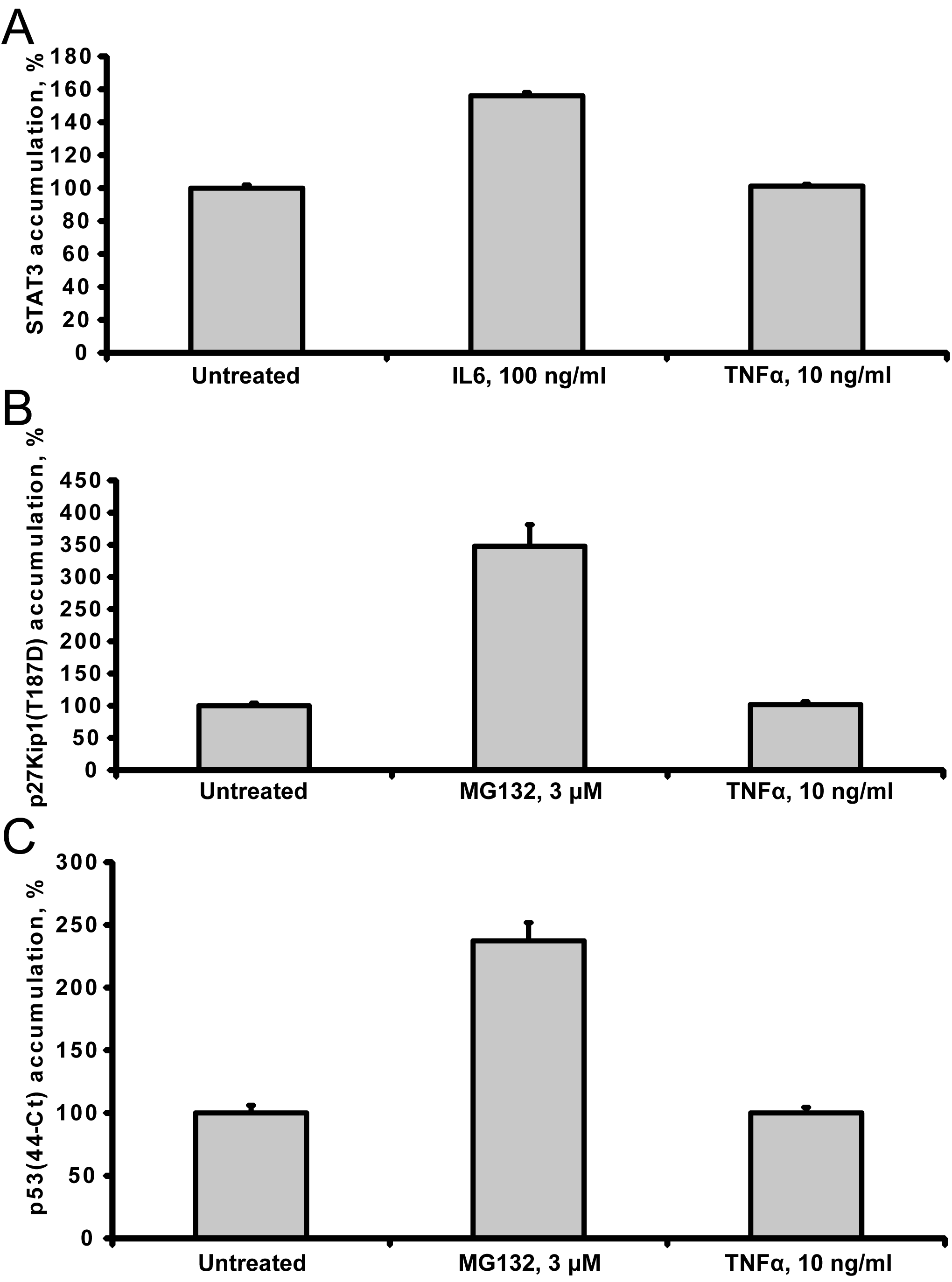

Supplement: Figure S7 — Effect of selected siRNAs on nuclear translocation of STAT3, degradation of p27, and degradation of p53 in U2OS osteosarcoma cells. Cells were incubated with TNFα (10 ng/mL) for 24 hr. Nuclear translocation of STAT3 (A), degradation of p27 (B), and degradation of p53 (C) were determined as described in Materials and Methods. All data (Median+/−MAD) normalized to cells transfected with control siRNA NTC1. (TIF) [file pone.0031270.s007.tif]

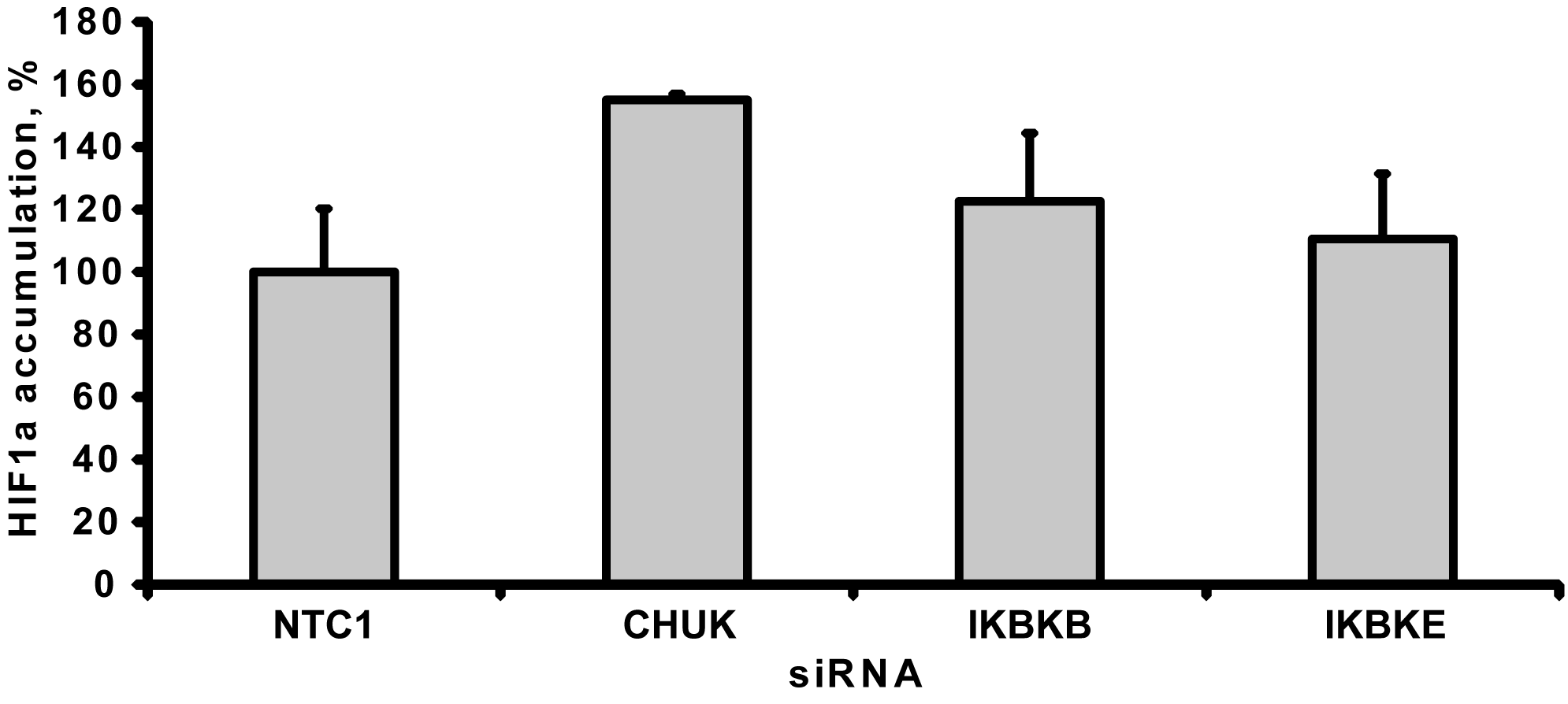

Supplement: Figure S8 — Effect of CHUK, IKBKB and IKBKE siRNAs on HIF1a accumulation in U2OS osteosarcoma cells. siRNA targeting CHUK, IKBKB and IKBKE were transfected into U2OS osteosarcoma cells. Cells were harvested 72 hr after transfection. Cells were treated with TNFα (10 ng/mL) for 24 hr before harvesting. All data normalized to untreated cells transfected with control siRNA NTC1. All data normalized to cells transfected with control siRNA NTC1. Data (Median+/−MAD) are representative of two independent experiments performed in triplicate. All data normalized to TNFα-treated cells. (TIF) [file pone.0031270.s008.tif]
